# A cross-sectional study of the association of dental health factors with progression and all-cause mortality in men diagnosed with HPV-associated oropharyngeal cancer

**DOI:** 10.1186/s12903-024-04047-6

**Published:** 2024-04-09

**Authors:** Brittney L. Dickey, L. Robert Gore, Robbert Slebos, Bradley Sirak, Kimberly A. Isaacs-Soriano, Kayoko Kennedy, Kristen Otto, J. Trad Wadsworth, Christine H. Chung, Anna R. Giuliano

**Affiliations:** 1 Center for Immunization and Infection Research in Cancer, H. Lee Moffitt Cancer Center and Research Institute, Tampa, FL USA; 2 Department of Cancer Epidemiology, H. Lee Moffitt Cancer Center and Research Institute, 12902 Magnolia Drive, Tampa, FL 33612 USA; 3 Biostatistics and Bioinformatics Shared Resource, H. Lee Moffitt Cancer and Research Institute, Tampa, FL USA; 4 Department of Head and Neck-Endocrine Oncology, H. Lee Moffitt Cancer and Research Institute, Tampa, FL USA; 5 Department of Biostatistics and Bioinformatics, H. Lee Moffitt Cancer and Research Institute, Tampa, FL USA

**Keywords:** Oropharyngeal cancer, HPV, Dental health

## Abstract

**Background:**

Human Papillomavirus-associated oropharyngeal cancer (HPV-OPC) incidence is increasing among men in the United States. Poor dental health has previously been associated with risk of head and neck cancers, oral HPV infection, and persistence but it is not understood whether dental health is associated with outcomes. We sought to determine the association of dental health with progression free survival and overall mortality among men with an HPV-OPC.

**Methods:**

A cross sectional study of men diagnosed with HPV-OPC between 2014–2020 at Moffitt Cancer Center in Tampa, FL was conducted. Dental records were abstracted for assessment of dental fitness prior to cancer treatment. Five dental factors including number of teeth lost, pocket depth, gingival score, loss of attachment, and bone loss were individually examined. Risk factor and outcome data were collected from a patient risk questionnaire and medical record. Using item response theory, an overall dental fitness score from five dental factors was developed in which missing data were multiply imputed. Cox proportional hazards model was used to assess whether dental factors were associated with progression-free survival or overall mortality.

**Results:**

Among 206 HPV-OPC cases, median follow-up was 3.4 years (IQR: 2.4–4.4) during which 40 cases involved progression or mortality and 25 deaths occurred. Overall dentition was significantly associated with progression free survival (*p* = 0.04) and with overall survival (*p* = 0.03) though findings were not significant after adjustment for age at diagnosis, stage, and smoking history (*p* = 0.146 and *p* = 0.120, respectively). A pocket depth of 7 mm or more was associated with overall survival (HR: 5.21; 95% CI: 1.43—19.11) and this remained significant after adjustment for confounding (aHR: 4.14; 95% CI: 1.72—16.26).

**Conclusions:**

Among men diagnosed with an HPV-associated OPC in the US, worse dental health was associated with reduced progression free survival and overall survival, but not after adjustment for confounders. Further studies are needed to examine whether dental health is associated with other prognostic factors and subsequent treatment-related outcomes.

## Background

A subset of head and neck cancers (HNC) that occur in the oropharynx is caused by oral infection with oncogenic types of human papillomavirus (HPV). In the United States, approximately 80% of oropharyngeal cancers (OPC, also known as oropharyngeal squamous cell carcinoma) are HPV-related, accounting for 40% of all HNC. The proportion of OPC attributable to HPV infection has increased in the recent past, as has the incidence of OPC, particularly among men [1, 2]. Patients diagnosed with HPV-OPC represent a unique population with distinguishable risk factors, treatment, and survival compared to OPC cases whose tumors are not HPV positive [3]. HPV-OPC, with incidence rates in the U.S. that now surpass that of cervical cancer incidence, has a better prognosis than HPV negative OPC [4]. As such, the diagnosis algorithm for OPC now includes testing for p16, a surrogate marker of HPV. p16 positive tumors are down staged as this is a strong prognostic biomarker [5, 6].

Survival after a diagnosis of HPV-OPC is better than among HPV negative cases although recurrence remains 13–25% within two years, and treatment is taxing regardless of tumor HPV status [7]. Radiation and surgery can be disfiguring and cause difficulty swallowing and long-term co-morbidities that are especially problematic in younger patients who often present with HPV-OPC [8]. It is recommended that all newly diagnosed HNC patients, including HPV-OPC, visit a dentist for an assessment of overall dental health prior to the start of cancer treatment. Poor dental health can increase the risk of osteoradionecrosis (ORN) that occurs with treatment [9]. As such, many patients prophylactically have several or most teeth extracted during this assessment to avoid ORN, although there is no consensus on what level of dental fitness is needed to avoid extensive extractions [10, 11].

In a prior study, HPV-OPC [12] cases were found to have a better overall dentition compared to HPV-negative OPC patients at the pre-radiotherapy dental assessment. However, other studies have found presence of periodontitis to be associated with OPC [13]. Further, HPV prevalence [14] and persistence [15], the precursor to OPC were also associated with periodontitis. Combined, these data indicate that components of dental fitness, including periodontal disease, may be associated with oral HPV persistence, subsequent risk of OPC, and poor treatment outcomes. A better understanding of the relationship between dental fitness and HPV-OPC outcomes is needed. This study aimed to assess the association between dental fitness of patients recently diagnosed with HPV-OPC and the treatment outcomes of cancer recurrence and death.

## Methods

### Study population

Male OPC cases 18 years and older were recruited from May 2014 to March 2020 from the Moffitt Cancer Center Head and Neck Cancer Radiation Oncology and Senior Adult Oncology clinics in Tampa, FL, US. Patients with a newly diagnosed oropharyngeal squamous cell carcinoma (C01.9 base of tongue; C05.1 soft palate, not otherwise specified [NOS]; C05.2 uvula; C09.0 tonsillar fossa; C09.1 tonsillar pillar; C09.8 overlapping lesion of the tonsil; C09.9 tonsil, NOS; C10.0 vallecula; C10.2 lateral wall of epiglottis; C10.3 posterior wall of epiglottis; C10.8 overlapping lesion of oropharynx and C10.9 oropharynx, NOS) were pre-screened for eligibility by reviewing medical records, then approached for study participation. Eligible patients met the criteria above and had not received any treatment for their new diagnosis. If men were interested in participating they provided informed consent prior to the start of any study procedures. Approval was obtained from Moffitt Cancer Center Scientific Review Committee and Institutional Review Board.

### Data collection

Participants completed a computer-assisted questionnaire that assessed demographic characteristics, personal and family cancer history, personal oral health and sexual behavior as well as alcohol, tobacco, and other drug use. Oral gargle specimens were collected from all study participants by swish-gargling with 15 mL of locally available mouthwash (Target Up&Up® [Water, alcohol (15% WT), glycerin,flavor, polysorbate 80, sodium saccharin, sodium benzoate, cetylpyridinium chloride, benzoic acid, blue 1, yellow 5) and returning to a collection vial [16].

HPV status in oral gargle samples and tumor tissues was ascertained. DNA was extracted from oral gargle cell pellets and a Formalin-Fixed Paraffin-Embedded (FFPE) tumor specimens using the automated BioRobot MDx (Qiagen). HPV status of oral gargle specimens was assessed using the HPV SPF_10_ PCR-DEIA-LiPA_25_ line probe assay (DDL Diagnostic Laboratory, Rijswik, the Netherlands) as previously published [17]. Immunostaining for p16^INK4a^ (p16), a surrogate marker of HPV presence in the tumor, was completed on FFPE samples and reviewed by qualified pathologists. For this study, participants were included if either tumor tissue was or p16 tested positive or DNA was positive for presence of HPV.

Participants provided the name of their dentist and gave written consent for staff to obtain data from their dental charts. Dental offices were contacted and asked to complete a record abstraction form pertaining to the last dental visit prior to the pathologically confirmed OPC diagnosis date (provided by our study team). If there was no exam prior to OPC diagnosis, abstraction was completed for the visit closest to OPC diagnosis, and those that occurred prior to any treatment were included in this study. From the dental records the following factors were assessed from the last dental office visit: total number of teeth lost, gingival index, pocket depth, presence of loss of attachment, and presence of alveolar bone loss.

Cancer relevant data including vital status, OPC recurrence, OPC subsite, clinical stage, and performance status were abstracted from participant medical records by clinicians with expertise in head and neck oncology for all years since OPC diagnosis up to March 2020. Date of death was recorded to ascertain survival time since diagnosis. Recurrence was recorded as binary with no date, as a recurrence date was often unavailable.

### Statistical analysis

The distribution of socio-demographic and dental health variables among participants was described. Because of small, expected sample sizes, Fisher exact testing was used to determine if any of the dental health indicators differed by smoking status, a factor known to be associated with both oral health and cancer. The associations between dental health and smoking were computed based on complete cases (pairwise) only. Each dental health indicator was modeled for its association with progression free survival (progression or mortality) and overall survival (all-cause mortality) using Cox proportional hazards models. Both were assessed in univariate models as well as multivariate models adjusted for age, stage, and smoking. Due to the high level of collinearity of the dental variables, they were not all entered in the models simultaneously. An alpha of 5% was considered significant and all analyses were completed using Stata 17.

As a result of prevalent missing dental data, multiple imputation was carried out in Stata 17, resulting in 20 datasets. Auxiliary variables were used to increase the plausibility of the missing at random (MAR) assumption. Because there is no automatic way to perform IRT in combination with multiple imputation, the imputations were first created, and then IRT was carried out separately for each imputed dataset. This resulted in an overall dental health score for each person in each imputed dataset. For analyses involving the imputed data, Stata automatically uses Rubins’ rules to combine results across imputations. With all dental variables, the mean values over 20 imputations were very close to the value obtained by dropping missing cases and computing the statistics on those that remained, thus supporting the validity of the imputation.

### Development of a dental score

An overall score of dental health was generated using item response theory (IRT) using guidelines from Raykov and Marcoulides [18]. Using IRT, for each participant, we estimated a continuous measure of dental risk using the selected dental health indicators, where each was dichotomized at its median. Gingival status was the variable with the highest rate of missingness (44.7%), a level considered not acceptable for IRT and so gingival status was dropped from the IRT analyses to develop an overall dental health score. Principal components analysis for each imputed dataset was used to ensure uniform variation across the dental health indicator variables. Next, due to small sample size, a single parameter IRT logistic model was fit to score each participant on the dental health variable and determine the threshold where each dental health indicator discriminated higher from lower dental health. The IRT model generated a continuous score, with a higher value indicating poorer dental health. The score was then categorized into tertiles to be entered into Cox models for progression free survival and overall survival.

## Results

Two hundred and thirty-one study participants who consented to dental record abstraction and for whom a completed dental abstraction form was received were included. After tumor HPV assessment 206 men were positive and included in this study. Median follow-up was 3.4 years (IQR: 2.4–4.4) with 15 cases of progression and 25 deaths. These HPV-positive OPC cases were mostly white (96.6%), non-Hispanic (96.6%), and with a median age of 62.0 years. Half of cases were never smokers and 45.9% were former smokers. Tumors occurred mostly in the tonsil (54.8%) or base of tongue (42.7%) and were stage 1 (96.1%) or lower using AJCC 8th edition staging criteria (Table 1).

**Table 1:** Sociodemographic and clinical factors of men with HPV-positive OPC (*n* = 206)

Characteristics	No	%^a^
**Race**		
White	198	96.6
Black	5	2.4
Other^b^	2	1.0
Refused	1	NA
**Ethnicity**		
Hispanic	7	3.4
Non-Hispanic	197	96.6
Refused	2	NA
**Age at OPC diagnosis**		
Median (range)	62.0 (38.8–87.5)	
35–49	18	8.7
50–59	70	34.0
60–69	73	35.4
≥ 70	45	21.8
**Marital status**		
Married/cohabiting	165	80.5
Single/divorced/separated/widowed	40	19.5
Refused or N/A	1	NA
**Education**		
High school (< 12 years)	48	23.5
Some college/vocational school	63	30.9
College graduate	62	30.4
Postgraduate/professional school	31	15.2
Refused or N/A	2	NA
**Smoking**		
Current	8	3.9
Former	94	45.9
Never	103	50.2
Refused or N/A	1	NA
**Alcohol drinks per occasion in the past month**		
None	72	35.5
1–4	107	52.7
≥ 5	24	11.8
Refused or N/A	3	NA
**Lifetime number of people kissed**		
None	3	1.5
1–9	43	21.8
10–24	57	28.9
25–49	41	20.8
≥ 50	53	26.9
Refused or N/A	9	NA
**Performed oral sex in the past 6 months**		
Yes	82	42.7
No	110	57.3
Refused or N/A	14	NA
**Tonsillectomy**		
Yes	88	42.9
No	117	57.1
Refused or N/A	1	NA
**Tumor location**		
Tonsil	113	54.8
BOT	88	42.7
Other OP	5	2.4
**Performance Status**		
ECOG 0/Karnofsky 100%	46	43.0
ECOG 1/Karnofsky 80–90%	55	51.4
ECOG 2/Karnofsky 60–70%	6	5.6
Unknown/Not Performed	99	NA
**Tumor Stage (AJCC 8th edition)**		
Stage I	137	66.5
Stage II	39	18.9
Stage III	22	10.7
Stage IV (A, B and C)	8	3.9
**HPV status in oral gargle**		
Positive	158	78.6
Negative	43	21.4
Missing	5	NA
**Number of teeth lost**		
0–4	70	34.8
5–7	60	29.9
8 +	71	35.3
Not Evaluated^c^	2	NA
Missing^d^	3	NA
**Gingival Index**		
0–1	62	54.9
2–3	51	45.1
Not Evaluated^c^	57	NA
Missing^d^	36	NA
**Pocket Depth**		
0-4 mm	81	47.1
4-6 mm	70	40.7
7 + mm	21	12.2
Not Evaluated^c^	22	NA
Missing^d^	12	NA
**Loss of attachment**		
Yes	95	65.1
No	51	34.9
Not Evaluated^c^	46	NA
Missing^d^	14	NA
**Alveolar Bone Loss**		
Yes	111	70.3
No	47	29.7
Not Evaluated^c^	36	NA
Missing^d^	12	NA

^a^Percentages calculated among patients with non-missing data

^b^Includes American Indian, Alaska Native, Native Hawaiian, Asian/Pacific Islander, and mixed race

^c^The dental provider indicated this was not evaluated at the visit date

^d^The dental provider did not provide a value

Dental variables were obtained from dental records between 4.6 years before diagnosis to 3.07 years after diagnosis. Among those with dental records before (*n* = 134) or on the same day as (*n* = 2) their OPC diagnosis, the mean (SD) of elapsed time was 233.0 (321.3) days and the median (IQR) was 122 (40.5–259) days. Among those with dental records after (*n* = 61) OPC diagnosis, mean (SD) of elapsed time was 126.5 (211.3) days and the median (IQR) was 28 (18–103) days.

Dental record abstraction revealed that of the patients with available data (i.e. not missing) 35.3% of patients had 8 or more teeth removed in their lifetime, 45.1% reported a higher gingival index (stage 2 or 3), and 12.2% with a pocket depth of 7 mm or greater. Loss of attachment and alveolar bone loss occurred in 65.1% and 70.3% of participants, respectively (Table 1).

Number of teeth lost (*p* = 0.001) and pocket depth (*p* = 0.025) was significantly different by smoking status (Table 2). A larger proportion of current smokers had lost eight or more teeth at the time of OPC diagnosis (62.5%) compared to 39.8% among former smokers and 27.5% among never smokers. More never smokers had pocket depth at or below 4 mm (50%) compared to former (45.3%) or current smokers (20%). Former smokers and current smokers had the highest proportion of men with a pocket depth greater than 7 mm (20.0%) compared to never smokers (5.6%).

**Table 2:** Association of smoking and dental factors

	**Smoking Status, No (Valid %)**			
	**Current** **(*n* =)**	**Former** **(*n* =)**	**Never** **(*n* =)**	*p*-value^*^
**Number of teeth lost**^a^				
0–4	1 (12.5)	20 (22.5)	49 (48.5)	**0.001**
5–7	2 (25.0)	32 (36.0)	24 (23.8)	
8 +	5 (62.5)	37 (41.6)	28 (27.7)	
Not evaluated	0	0	0	NA
Missing	0	4	1	
**Gingival index**				
0–1	1 (25.0)	30 (58.8)	29 (51.8)	0.404
2–3	3 (75.0)	21 (41.2)	27 (48.2)	
Not evaluated	4	22	30	NA
Missing	0	20	16	
**Pocket depth**				
0–4 mm	1 (20.0)	34 (45.3)	45 (50.0)	**0.025**
4–6 mm	3 (60.0)	26 (34.7)	40 (44.4)	
7 + mm	1 (20.0)	15 (20.0)	5 (5.6)	
Not evaluated	3	9	10	NA
Missing	0	9	2	
**Loss of attachment**				
Yes	2 (33.3)	21 (32.8)	26 (35.6)	0.95
No	4 (66.7)	43 (67.2)	47 (64.4)	
Not evaluated	2	21	23	NA
Missing	0	8	6	
**Alveolar bone loss**				
Yes	3 (37.5)	16 (23.5)	26 (32.9)	0.37
No	5 (62.5)	52 (76.5)	53 (67.1)	
Not evaluated	0	18	18	
Missing	0	7	5	
**Dental score (tertile of IRT derived score)**				
Lowest	3 (37.5)	28 (30.1)	33 (32.4)	**0.035**
Middle	1 (12.5)	31 (33.3)	48 (47.1)	
Highest	4 (50.0)	34 (36.6)	21 (20.6)	

^a^Excludes 3 patients who were missing data on smoking status

*Fishers exact testing

Dental factors and the overall dental fitness score were assessed for association with progression free survival (progression or mortality) in Table 3. In tests for trend, pocket depth (*p* = 0.035) and the dental score (*p* = 0.040) were significantly associated with increased risk of OPC progression or mortality in unadjusted analyses. The highest category of pocket depth (7 mm or greater) was associated with greater risk of progression or mortality (HR: 2.62; 95% CI: 1.03—6.72). No dental risk variable was associated with progression free survival after adjusting for the confounders age, stage, and smoking.

**Table 3:** Dental factors and risk of progression or mortality (*n* = 40)

	**Event No. (Valid %)**	**HR (95% CI)**	**P**_**trend**_	**aOR (95% CI)**^**a**^	**P**_**trend**_
**Number of teeth lost**					
0–4	10 (26.3)	REF	0.058	REF	0.194
5–7	10 (26.3)	1.03 (0.42, 2.54)		0.95 (0.36, 2.49)	
8+	18 (47.4)	2.03 (0.94, 4.39)		1.64 (0.71, 3.79)	
**Gingival index**					
0–1	5 (33.3)	REF	0.273	REF	0.534
2–3	10 (66.7)	1.57 (0.70, 3.55)		1.32 (0.54, 5.27)	
**Pocket depth**					
<4 mm	8 (28.6)	REF	**0.035**	REF	0.100
4–6 mm	13 (46.4)	1.73 (0.78, 3.85)		1.46 (0.64, 3.33)	
>7 mm	7 (25.0)	**2.62 (1.03, 6.72)**		2.33 (0.84, 6.42)	
**Loss of attachment**					
No	5 (19.2)	REF	0.204	REF	0.438
Yes	21 (80.8)	1.76 (0.74, 4.21)		1.45 (0.57, 3.69)	
**Alveolar bone loss**					
No	4 (13.8)	REF	0.158	REF	0.314
**Yes**	25 (86.2)	2.02 (0.76, 5.36)		1.69 (0.60, 4.74)	
**Dental score**					
Lowest	7 (17.5)	REF	**0.040**	REF	0.146
Middle	15 (37.5)	1.75 (0.58, 5.25)		1.62 (0.52, 5.02)	
Highest	18 (45.0)	2.70 (1.0, 7.40)		2.13 (0.73, 6.22)	

^a^Adjusted for age, stage, and smoking cox proportional hazards model for progression free survival (progression or mortality)

In Table 4, dental factors and the dental score were investigated for their association with all-cause mortality. In tests for trend, pocket depth (*p* = 0.010) and the dental score (*p* = 0.030) were significantly associated with increased risk of mortality in unadjusted analyses. Interestingly, all deaths occurred in OPC cases that had attachment loss and thus the hazard ratio was not estimable. The highest category of pocket depth (7 mm or greater) was associated with greater risk of mortality in unadjusted (HR: 5.21; 95% CI: 1.43 – 19.11) and adjusted (aHR: 4.14; 95% CI: 1.72 – 16.26) analyses. Dental score was no longer significantly associated with mortality in adjusted analyses.

**Table 4:** Dental factors and risk of mortality (*n* = 25 deaths)

	**Event No. (Valid %)**	**HR (95% CI)**	**P**_**trend**_	**aOR (95% CI)**^**a**^	**P**_**trend**_
**Number of teeth lost**					
0–4	5 (21.7)	NA	0.073	NA	0.325
5–7	6 (26.1)	1.35 (0.41, 4.43)		1.26 (0.35, 4.56)	
8 +	12 (52.2)	2.50 (0.88, 7.14)		1.75 (0.55, 5.53)	
**Gingival index**					
0–1	1 (11.1)	NA	0.165	NA	0.320
2–3	8 (88.9)	2.31 (0.70, 7.56)		1.96 (0.51, 7.46)	
**Pocket depth**					
< 4 mm	3 (16.7)	NA	**0.010**	NA	**0.042**
4–6 mm	9 (50.0)	2.34 (0.67, 8.17)		1.89 (0.51, 6.96)	
> 7 mm	6 (33.3)	**5.21 (1.43, 19.11)**		**4.14 (1.72, 16.26)**	
**Loss of attachment**					
No	0 (0.0)	NA	NA	NA	NA
Yes	15 (100.0)	NA		NA	
**Alveolar bone loss**					
No	1 (6.2)	NA	0.179	NA	0.322
Yes	15 (93.8)	3.11 (0.59, 16.43)		2.41 (0.42, 13.89)	
**Dental score**					
Lowest	2 (8.0)	NA	**0.030**	NA	0.120
Middle	10 (40.0)	3.06 (0.58, 16.07)		2.96 (0.54, 16.26)	
Highest	13 (52.0)	4.80 (0.93, 24.68)		3.60 (0.63, 20.64)	

^a^Adjusted for age, stage, and smoking cox proportional hazards model for overall survival (all-cause mortality)

## Discussion

This is the first study to investigate the association between dental fitness and cancer treatment outcomes of progression and death, among men diagnosed with HPV-related OPC. A worse overall dental health score was not significantly associated with higher odds of OPC progression or death or all-cause mortality in analyses adjusted for age, stage, and smoking. Pocket depth alone was significantly associated with risk of progression or death in men diagnosed with HPV-OPC. For overall survival (but not progression free survival) this held after adjusting for age, stage, and smoking status.

Dental health is assessed among patients diagnosed with HNC to inform prevention of radiotherapy related complication and identification of poor dental health can lead to prophylactic removal of several teeth in an effort to prevent osteoradionecrosis. Recent studies from Patel et al. found that several subsites of HNC [19] and patients with OPC, specifically, had better dental fitness than other HNC patients [20]. However, osteoradionecrosis is more likely to occur among patients diagnosed with OPC compared to other subsites due to the radiation coverage to the jaw [21]. A prior study found HPV-positive OPC patients had significantly more teeth, more restored teeth, less tooth decay, and less severe bone loss compared to HPV-negative OPC patients indicating better overall dental health management but leaving more teeth vulnerable to post-treatment complications [9]. Our study focused on HPV-positive OPC as they represent over 90% of the OPC patients seen at our institution. Of these patients, the majority had dental records available for abstraction with recent dental exams. This may reflect a pattern of more regular visits to the dentist and better dental management among men with HPV-OPC in our study. In our study population, 60% of men had lost > 5 teeth, and approximately half the men had experienced the more severe dental conditions of loss of attachment (46.%) or alveolar bone loss (54%).

Differences in smoking, age, and dental engagement may influence disparity in dental care by HPV status. Smoking has been consistently associated with head and neck cancer and poor oral health. Similarly, in this study, smoking was significantly associated with number of teeth lost and pocket depth. Therefore, it was essential to control for smoking, when assessing associations between dental health and HPV-OPC outcomes. HPV-associated OPC cases are also different than their HPV-negative counterparts with respect to age at diagnosis. The median age of HPV-OPC cases is 5 years younger than HPV-negative OPC patients [1, 2]. In a study of HPV-associated OPC patients, increasing age and current or previous smoking history was associated with worse dental fitness, but this diminished when stratified by HPV status [9].

Periodontal disease is an irreversible damage of the tissues surrounding the teeth measured by gum pockets and alveolar bone loss. Periodontitis has been associated with an increase in release of bacteria and inflammatory markers into the saliva [22]. Limited evidence suggests periodontal disease may be a risk factor for oral HPV infection [23]. It is thought that the dysbiosis of the oral microbiota leaves a person susceptible to HPV acquisition and integration via the exposed oral mucosal reservoir caused by abrasions from chronic inflammation [22, 23]. Periodontal disease has been implicated in risk of treatment comorbidities among OPC patients. A prior study of 266 HNC patients and 207 cancer-free controls found a one millimeter increase in alveolar bone loss was associated with more than a fourfold increase in risk of HNC, with risk greatest among oral cavity cases. [13] In another study of 30 tongue squamous cell carcinoma tumor cases, alveolar bone loss, but not teeth loss was associated with increased odds of HPV positivity in the tumor [22]. However, these studies stop short of exploring how periodontal disease may also be related to response to cancer treatment. Our study, though, observed an association between worse dental health with recurrence among treated HPV-OPC patients, but the biologic mechanism underlying this observation remains unclear.

This study leveraged data abstracted from dental charts to investigate dental factors associated with treatment outcomes among patients diagnosed with HPV-OPC. As with any observational study there are limitations that should be noted. First, our study was specifically conducted among men with HPV-related OPC. As the majority of OPC cases diagnosed in the US are among men, though, this study included the leading at-risk group and largest proportion of OPC cases. The men included in this study are representative of most new cases of HPV-OPC diagnosed in the US. Since the study was conducted solely among OPC patients who were tumor HPV-positive, results cannot be directly compared to an HPV-negative group and generalizability to women should be limited. There are strengths also worth noting. First, the dental health variables were assessed via record abstraction as opposed to self-report (which may lead to recall bias). Second, the dental health score created in this study, based as it is on multiply imputed data to fill in for missingness, presented a novel approach to overall dental fitness when some participants did not have data on all dental health variables. Finally, a relatively large sample size of HPV-OPC cases with dental data available followed for a median of 3.4 years allowed for robust statistical analyses adjusting for key confounders. Future studies should investigate dental factors among HPV-negative OPC patients, including women, to determine differences in OPC outcomes associated with oral health by HPV status and differences by gender.

Overall, this study of outcomes in men diagnosed with HPV-OPC found worse overall dental health to be associated with worse progression, but attenuated when adjusted for other factors associated with poor outcomes (age, stage, smoking). Greater pocket depth was associated with increased risk of death after cancer diagnosis after controlling for age, stage, and smoking status. Furthermore, all deaths were observed among individuals with loss of alveolar attachment. The results of this study indicate a need for a thorough evaluation of dental health in men diagnosed with HPV-OPC to not only prevent treatment related complications, but also improve outcomes after diagnosis.

## Data Availability

The datasets analyzed during the current study are not publicly available to protect participant confidentiality but are available from the corresponding author on reasonable request.
